# Autoimmune Thyroiditis Mitigates the Effect of Metformin on Plasma Prolactin Concentration in Men with Drug-Induced Hyperprolactinemia

**DOI:** 10.3390/ph17080976

**Published:** 2024-07-23

**Authors:** Robert Krysiak, Marcin Basiak, Witold Szkróbka, Bogusław Okopień

**Affiliations:** Department of Internal Medicine and Clinical Pharmacology, Medical University of Silesia, Medyków 18, 40-752 Katowice, Poland; mbasiak@sum.edu.pl (M.B.); wszkrobka@sum.edu.pl (W.S.); bokopien@sum.edu.pl (B.O.)

**Keywords:** autoimmune thyroid disease, insulin sensitivity, lactotropes, men, prolactin excess

## Abstract

Metformin inhibits the secretory function of overactive anterior pituitary cells, including lactotropes. In women of childbearing age, this effect was absent if they had coexisting autoimmune (Hashimoto) thyroiditis. The current study was aimed at investigating whether autoimmune thyroiditis modulates the impact of metformin on the plasma prolactin concentration in men. This prospective cohort study included two groups of middle-aged or elderly men with drug-induced hyperprolactinemia, namely subjects with concomitant Hashimoto thyroiditis (group A) and subjects with normal thyroid function (group B), who were matched for baseline prolactin concentration and insulin sensitivity. Titers of thyroid peroxidase and thyroglobulin antibodies, levels of C-reactive protein, markers of glucose homeostasis, concentrations of pituitary hormones (prolactin, thyrotropin, gonadotropins, and adrenocorticotropic hormone), free thyroxine, free triiodothyronine, testosterone, and insulin growth factor-1 were measured before and six months after treatment with metformin. Both study groups differed in titers of both antibodies and concentrations of C-reactive protein. The drug reduced the total and monomeric prolactin concentration only in group B, and the impact on prolactin correlated with the improvement in insulin sensitivity and systemic inflammation. There were no differences between the follow-up and baseline levels of the remaining hormones. The results allow us to conclude that autoimmune thyroiditis mitigates the impact of metformin on prolactin secretion in men.

## 1. Introduction

Despite maintaining anatomical and functional connections with the brain, the pituitary is situated outside the blood–brain barrier [1]. This fact may explain why metformin, a drug of unquestionable importance in the treatment of type-2 diabetes and other insulin-resistant states [2], preferentially accumulates in this structure [3] and attenuates the secretory function of different anterior pituitary cells, including thyrotropes [4,5], gonadotropes [6,7], and lactotropes [8,9,10,11,12]. Interestingly, and probably importantly from the clinical point of view, the inhibitory effect on thyroid-stimulating hormone (TSH), follicle-stimulating hormone (FSH), luteinizing hormone (LH), and prolactin in these studies was observed only if their baseline levels were elevated. There is no evidence that metformin administration, even at high doses, results in a deficiency of pituitary hormones. The decrease in prolactin secretion has been reported irrespective of the reason for prolactin excess, including in subjects with prolactinomas [8], empty sella syndrome [8], traumatic brain injury [8], drug-induced hyperprolactinemia [9,10,11,12], and in hyperprolactinemia of unknown origin [8]. Unlike most cases of prolactin excess, which may be effectively treated with dopamine agonists (mainly bromocriptine and cabergoline), the use of these agents in antipsychotics-induced hyperprolactinemia is much more controversial because they may aggravate psychiatric disability [13]. Considering doubts concerning the use of dopaminergic agents and the functional basis of this disorder, metformin is regarded by some research groups as a potential, emerging treatment for drug-induced prolactin excess [14]. It should be kept in mind that even moderate long-term prolactin excess, irrespective of gender, predisposes to insulin resistance, prediabetes, atherogenic dyslipidemia, excessive and/or subnormal fat accumulation, subclinical atherosclerosis, and endothelial dysfunction [15,16,17,18,19,20], and therefore, it should be avoided. It seems that metformin is characterized by differences in the action of lactotrope function in different populations of subjects with hyperprolactinemia. The impact of this agent on circulating prolactin levels was found to be determined by sex because in young adults the drug reduced prolactin levels only in women but not in men [21]. Moreover, metformin reduces prolactin levels in men if they have low, but not if they have normal, testosterone concentrations [22]. These discrepancies suggest that the impact of metformin on prolactin secretion may be more prominent in individuals with low testosterone production. Testosterone deficiency may make patients with prolactin excess particularly prone to cardiometabolic complications because low testosterone production in men is often associated with metabolic comorbidities, such as obesity, insulin resistance, metabolic syndrome, and type-2 diabetes mellitus [23,24].

Recently, our research team reported that coexisting autoimmune (Hashimoto) thyroiditis mitigated the impact of metformin on the secretory function of the anterior pituitary cells of women, including attenuation of the prolactin-lowering effect [25,26]. This finding seems to be clinically relevant because Hashimoto thyroiditis is one of the most common human disorders and the most prevalent organ-specific autoimmune disorder worldwide [27,28]. Despite the female preponderance of autoimmune thyroiditis, more and more males are being diagnosed with this clinical entity [29]. To the best of our knowledge, no previous study investigated the association between metformin and autoimmune thyroiditis at the level of male lactotropes. Few studies conducted so far suggest that low testosterone concentrations and autoimmune thyroiditis are, in men, reciprocally related. Males with primary hypothyroidism, caused in the majority of patients by autoimmune thyroid disease, were characterized by gonadal hypofunction [30]. A higher ratio of estradiol to testosterone in males was associated with autoimmune thyroid disease [31]. Shorter AR (CAG)n repeats, increasing the activity of the androgen receptor, were found to predispose to a younger onset of autoimmune thyroid disease [32]. Moreover, Hashimoto thyroiditis is highly prevalent in individuals with Klinefelter’s syndrome, the most prevalent sex-chromosome disorder resulting in male hypogonadism [33]. Lastly, exogenous testosterone reduced thyroid antibody titers in euthyroid men with testosterone deficiency and Hashimoto thyroiditis [34]. In addition to the association with testosterone deficiency, particularly in individuals with markedly elevated prolactin levels [35]; increased prolactin production may predispose to the development and progression of autoimmune disorders, including autoimmune thyroiditis [36]. Thus, the present study investigated whether coexisting euthyroid Hashimoto thyroiditis modulates the impact of chronic metformin treatment on plasma prolactin levels in middle-aged and elderly men with antipsychotic-induced hyperprolactinemia.

## 2. Results

The study groups were comparable with respect to age, smoking, percentages of patients with type-2 diabetes and prediabetes, BMI, and blood pressure (systolic and diastolic) (Table 1). Titers of thyroid antibodies (both TPOAb and TgAb, and the concentration of hsCRP were higher in group A than in group B. Levels of glucose, glycated hemoglobin, pituitary hormones (prolactin [total and monomeric], TSH, FSH, LH and ACTH, macroprolactin), free thyroid hormones, testosterone, IGF-1, and HOMA-IR, did not differ between groups A and B (Table 2).

Metformin treatment was well tolerated; side effects (decreased appetite, transient diarrhea, tiredness, and weakness) were mild and present only in the minority of patients (12.5% in group A and 8.3% in group B). Because no patient prematurely discontinued the treatment, the data of all included individuals were statistically analyzed. Over the entire study period, all patients adhered to the treatment recommendations and the recommendations concerning diet and physical activity. 

Metformin did not affect BMI (group A: 24.9 ± 4.3 kg/m^2^ vs. 24.3 ± 4.6 kg/m^2^ [*p* = 0.6428], group B: 24.5 ± 4.8 kg/m^2^ vs. 23.9 ± 4.1 kg/m^2^ [*p* = 0.6437]), and follow-up BMI did not differ between both groups (*p* = 0.7519). In both treatment groups, metformin decreased glucose, HOMA-IR, and glycated hemoglobin. In group B, but not in group A, there were differences between the baseline and the follow-up plasma concentrations of both total and monomeric prolactin and in hsCRP. Titers of TPOAb and TgAb, concentrations of macroprolactin, TSH, free thyroxine, free triiodothyronine, gonadotropins, testosterone, ACTH, and IGF-1 remained at similar levels over the entire study period. At the end of the study, there were between-group differences in glucose, HOMA-IR, glycated hemoglobin, total prolactin, monomeric prolactin, antibody titers, and hsCRP (Table 2). 

The study groups differed in the percent changes from baseline for glucose, HOMA-IR, glycated hemoglobin, total prolactin, monomeric prolactin, testosterone, and hsCRP, which were greater in group B than in group A (Table 3).

At the beginning of the study, in group A, there were positive correlations between the hsCRP concentration and the antibody titers (TPOAb: r = 0.462, *p* = 0.0002; TgAb: r = 0.398, *p* = 0.0011). The effect of treatment on the total and monomeric prolactin correlated with the (a) baseline concentrations (total prolactin—group A: r = 0.411, *p* = 0.0008, group B: r = 0.325, *p* = 0.0322; monomeric prolactin—group A: r = 0.442, *p* = 0.0004, group B: r = 0.346, *p* = 0.0226), (b) the impact of treatment on HOMA-IR (total prolactin—group A: r = 0.368, *p* = 0.0282, group B: r = 0.340, *p* = 0.0226; monomeric prolactin—group A: r = 0.394, *p* = 0.0014, group B: r = 0.411, *p* = 0.0008), and (c) in group B additionally, also with treatment-induced reduction in hsCRP concentration (r = 0.365, *p* = 0.0122 for total prolactin; r = 0.385, *p* = 0.0043 for monomeric prolactin). In group A, there were inverse correlations between the baseline antibody titers and metformin action on total prolactin (r = −0.402, *p* = 0.0005 for TPOAb; r = −0.375, *p* = 0.0049 for TgAb) and on monomeric prolactin (r = −0.387, *p* = 0.0023 for TPOAb; r = −0.341, *p* = 0.0274 for TgAb), as well as between treatment-induced changes in HOMA-IR and the baseline hsCRP (r = −0.352, *p* = 0.0126). In group B, treatment-induced changes in prolactin correlated with the impact of treatment on LH (r = 0.315, *p* = 0.0312 for total prolactin; r = 0.342, *p* = 0.0265 for monomeric prolactin) and inversely with the baseline testosterone (r = −0.265, *p* = 0.0489 for total prolactin; r = −0.269, *p* = 0.0411 for monomeric prolactin). The remaining correlations were insignificant.

## 3. Discussion

The study showed a decrease in prolactin concentrations in men aged between 50 and 75 years with elevated levels of this hormone who do not have concurrent thyroid disease. This finding contrasts with our previous observations concerning a neutral effect of metformin on prolactin levels in a younger population of hyperprolactinemic men [21]. This incongruency may be well explained by lower mean testosterone concentrations in the participants of the current study. In line with this interpretation, the reduction in the prolactin concentration is inversely correlated with the plasma testosterone concentration. Another interesting finding is that the decrease in the total prolactin level reflected a reduction in the monomeric form of prolactin. In turn, the concentration of macroprolactin, high molecular mass forms of this hormone, composed of antigen–antibody complexes of prolactin and aggregates of covalent or noncovalent polymers of monomeric prolactin [37], remained unchanged, which has been evidenced so far only in women [38]. The obtained results indicate that metformin treatment should be considered in middle-aged elderly men with diabetes or prediabetes coexisting with an elevated prolactin concentration. Because of the cardiometabolic and bone consequences of long-term prolactin excess, the risk of which increases with age [39], metformin treatment seems to bring benefits even in men with asymptomatic hyperprolactinemia. Another argument in favor of metformin is that antipsychotics seem to increase the risk of developing diabetes [40], the presence of which additionally justifies this treatment. It is worth noting that metformin treatment was well tolerated by men receiving antipsychotic drugs and did not worsen the effectiveness of antipsychotic therapy.

Metformin treatment did not significantly affect gonadotropin levels, though both groups differed in the strength of the prolactin-lowering effect, and in men without thyroid pathology, the decrease in plasma prolactin positively correlated with the impact of treatment on LH. This finding may suggest that metformin exerted a weak, and statistically insignificant, inhibitory effect on gonadotropin secretion in this population. The study included men with only mild or moderate prolactin excess but not individuals with severe hyperprolactinemia, which is often complicated by gonadal failure [41]. Thus, a relatively low mean baseline testosterone concentration (though in the majority of patients still within the reference range) was probably a consequence of age-related changes in testosterone production (late-onset hypogonadism), while the association with prolactin excess was less convincing. This explains why the improvement in lactotrope function did not result in statistically significant changes in gonadotropins and testosterone.

Undoubtedly, a novel observation of our present study is that the impact on prolactin concentration was absent in men with concurrent euthyroid Hashimoto thyroiditis. Although autoimmune thyroiditis is the major cause of hypothyroidism in developed countries, less pronounced lymphocytic infiltration and fibrotic reactions do not result in thyroid hypofunction [42]. Consequently, euthyroid Hashimoto thyroiditis is considered a milder form of autoimmune thyroiditis than hypothyroid Hashimoto thyroiditis and often precedes the hypothyroid phase [43]. Because untreated or inadequately treated hypothyroidism often causes hyperprolactinemia [44], making it difficult to differentiate prolactin excess secondary to thyroid hypofunction and antipsychotic treatment, the study included only patients with normal concentrations of TSH, free thyroxine, and free triiodothyronine, which were suggestive of an intact hypothalamic–pituitary–thyroid axis activity. Another reason for including only euthyroid men is the link between schizophrenia and hypothyroidism resulting in that all patients receiving antipsychotics on a chronic basis should be effectively substituted with thyroid hormones [45]. Thus, our findings cannot be explained by thyroid hypofunction. Moreover, because of matching, they cannot be attributed to baseline differences in prolactin concentration and insulin sensitivity. Lastly, they cannot be explained by differences in testosterone production, which was similar in both study groups, as well as by other differences between the groups. Thus, attenuation of the prolactin-lowering effect of metformin was likely associated with thyroid autoimmunity. In line with this explanation, titers of TPOAb and TgAb, which determine the severity of Hashimoto thyroiditis [28], inversely correlated with the impact of metformin on total and monomeric prolactin. Our finding is consistent with similar observations in women [25,26], which suggest that the pituitary effects of metformin are mitigated by Hashimoto thyroiditis, irrespective of gender.

In the investigated population of middle-aged and elderly men, metformin did not affect thyroid antibody titers. This finding is in disagreement with the results of a meta-analysis of four clinical studies [46], which showed a reduction in both TPOAb and TgAb. There are different possible explanations for these contradictory results. They may be attributed to differences in the assessed populations because the meta-analysis by Jia et al. included mainly women of reproductive age (the percentage of men was very low). Second, a remarkable proportion of the analyzed patients was diagnosed with subclinical hypothyroidism. Thus, the degree of inflammation and the destruction of thyrocytes was greater than in the current study. Finally, we recruited a smaller number of patients in comparison with the number of individuals included in the mentioned Chinese meta-analysis. A neutral effect on thyroid antibody titers indicates that the weak effects of metformin on prolactin levels in patients with autoimmune thyroiditis cannot be explained by a direct involvement of these antibodies. 

Another finding attracting attention is the positive correlations between the impact of treatment on prolactin and insulin sensitivity, indicating that the metabolic and prolactin-lowering effects of metformin are related to each other, supporting the view that prolactin plays an important role in glucose homeostasis [15,16,17,18,19,20]. More importantly, concomitant autoimmune thyroiditis weakened metformin action on glucose homeostasis, which is supported by inverse correlations between the impact on HOMA-IR and hsCRP concentrations. Though our study was not aimed at investigating molecular mechanisms of metformin action, the obtained results may suggest that relatively small changes in plasma glucose, glycated hemoglobin, and HOMA-IR resulted from markedly elevated post-treatment prolactin levels in this population and/or from interaction between proinflammatory markers and metformin at the level of the central nervous system and/or GLUT-4, the main insulin-responsive glucose transporter, which is a key regulator of systemic glucose homeostasis [25,26,47].

The most likely explanation for a mitigatory impact of Hashimoto thyroiditis on the prolactin-lowering effect of metformin is interaction at the level of the pituitary adenosine 5′-monophosphate-activated protein kinase (AMPK) pathway. Some findings support this explanation. First, activation of AMPK is one of the main mechanisms of metformin action [48]. Second, anterior pituitary cells are characterized by an abundant expression of this enzyme [49]. Third, pituitary AMPK was found to mediate the gonadotropin-lowering effects of metformin [49], and it may mediate other pituitary effects of this drug. Lastly, hsCRP and proinflammatory cytokines, found to be overproduced in Hashimoto thyroiditis [28], exert an inhibitory effect on the activity of the AMPK pathway [50,51]. Thus, it is likely that the stimulatory effect observed in men without thyroid autoimmunity is counterbalanced by the opposite effect of the proinflammatory state. This interaction may be particularly relevant in middle-age or elderly men because low testosterone production, characterizing men in these age groups, is associated with the low activity of AMPK [52]. The alternative explanation for our findings is the opposite impact of metformin and mediators of the proinflammatory state on the activity of tuberoinfundibular dopaminergic neurons, playing a fundamental role in the regulation of prolactin secretion [53]. In line with this explanation, metformin was found to increase the central dopaminergic tone [54], where proinflammatory factors inhibited the tuberoinfundibular dopaminergic system [55]. Interestingly, increased activity of both AMPK and tuberoinfundibular dopaminergic neurons is associated with increased insulin sensitivity [56,57] and may explain correlations between the impact of metformin on prolactin and HOMA-IR. 

Some other conclusions can be drawn on the basis of the obtained results. First, autoimmune thyroid disease attenuates the metabolic effects of metformin in subjects without hypothyroidism. Thus, men with Hashimoto thyroiditis and either diabetes or at high diabetes risk may gain fewer benefits from metformin treatment than their peers without thyroid disease. This finding is worth underlying because euthyroid Hashimoto thyroiditis may be complicated by insulin resistance and may predispose to type-2 diabetes and prediabetes [58]. Second, in the current study, metformin was administered at a high dose (3 g daily). In our previous ones, metformin decreased prolactin levels only if the daily dose ranged from 2.55 to 3 g [8,12]. Thus, only high-dose metformin should be recommended to reduce prolactin levels in subjects with prolactin excess. However, high doses of this agent are well tolerated even in the case of prediabetes. Finally, positive correlations between changes in total and monomeric prolactin and their baseline concentrations, as well as no statistically significant changes in the concentrations of the remaining hormones, the baseline levels of which were within the reference range, suggest that metformin does not lead to hypoprolactinemia and other pituitary hormone deficiencies. This conclusion is clinically relevant because dopaminergic agents may cause prolactin deficiency, which, at least in premenopausal women, is associated with the unfavorable metabolic profile [59].

Our study provides some therapeutic implications. First, it seems justified to assess thyroid antibodies in all men requiring antipsychotic therapy. Finding autoimmune thyroiditis should be an argument against treatment with first-generation antipsychotics, amisulpride, risperidone, and paliperidone, particularly predisposing to hyperprolactinemia [60]. Second, measurement of thyroid antibodies may be considered in all insulin-resistant men poorly responding to metformin therapy, if the reason for this resistance is unknown. Third, metformin may be considered as a treatment option for antipsychotic-induced hyperprolactinemia in men without thyroid disease if other options are not available, effective, or tolerated. However, its administration should not be recommended for patients with concomitant thyroid autoimmunity. Fourth, the treatment of patients with both prolactin excess and thyroiditis may be very difficult because the prolactin-lowering effects of cabergoline in this group are weak [61]. Last, from a pathophysiological point of view, men with diabetes or prediabetes may benefit from concomitant treatment with metformin and agents reducing thyroid antibody titers, such as vitamin D, selenomethionine, and myo-inositol [62]. Certainly, it cannot be excluded that other insulin-sensitizing agents, such as dipetidyl peptidase-4 inhibitors, glucagon-like peptide-1 agonists, and thiazolidinediones, administered in monotherapy or in combination with metformin, are superior to metformin alone in patients with autoimmune thyroiditis.

The strength of our study was a homogenous population of patients, resulting from strict inclusion and exclusion criteria. Unfortunately, this causes a situation where we can only speculate about metformin action in other groups of patients, particularly individuals with severe hyperprolactinemia or hypothyroidism. For ethical reasons, we did not include men with prolactin levels above 80 ng/mL. Severe hyperprolactinemia must always be specifically addressed, even when there is no sexual dysfunction (reduced libido and/or importance) because of the medium-/long-term risk of osteoporosis and cardiovascular issues [63]. Such patients require specific treatment, including the discontinuation or decreasing the dose of the antipsychotic drug, changing the antipsychotic drug, the addition of aripiprazole, the addition of dopamine agonists, and/or symptomatic treatment) [60]. The obtained correlations (positive between metformin action on plasma prolactin and baseline prolactin levels and negative between the impact on plasma prolactin and thyroid antibody titers) suggest, however, that metformin may reduce prolactin levels also in patients with severe hyperprolactinemia, but that this effect is probably also attenuated by coexisting autoimmune thyroiditis. Moreover, we excluded not only levothyroxine-naive patients with overt or subclinical hypothyroidism but also hypothyroid individuals in whom euthyroidism was restored by levothyroxine substitution. This enabled us to eliminate possible pharmacokinetic and pharmacodynamic interactions between metformin and exogenous levothyroxine. The results of our recent study [64] indicate that metformin reduces TSH in patients with autoimmune thyroiditis-induced hypothyroidism. However, considering that the impact of this agent on TSH in hypothyroid patients was moderate, even if metformin reduces plasma prolactin in hypothyroid men with autoimmune thyroiditis and antipsychotics-induced hyperprolactinemia, this effect is probably weak and secondary to the improvement in the hypothalamic–pituitary–thyroid axis activity but not associated with a direct effect on lactotrophic cells and/or the tuberoinfundibular dopaminergic pathway.

There are several other study limitations that should be noted. Despite being adequately powered for the primary outcome, the small sample size limits the generalizability of the results. Owing to the study design, the results might have been potentially affected by selection and confounding bias. Extrapolating previous data [65,66], it seems that the study population was characterized by sufficient iodine and insufficient selenium intake. It cannot completely exclude some differences in the impact of autoimmune thyroiditis in men with inadequate iodine and/or adequate selenium intake. Strict inclusion and exclusion criteria do not allow us to conclude whether other autoimmune disorders (including Graves’ disease) modulate metformin action on antipsychotic-induced hyperprolactinemia, which justifies further studies. Precautions during study design and data analysis limited, but did not completely eliminate, the regression toward the mean [67]. Lastly, because gel-filtration chromatography is time-consuming and expensive, the macroprolactin content was measured using a less accurate polyethylene glycol precipitation method [37].

In conclusion, chronic treatment with metformin decreases elevated prolactin levels in middle-aged and elderly men receiving antipsychotic therapy. The inhibitory effect of this drug on lactotrope secretory function is not observed if hyperprolactinemia is accompanied by Hashimoto thyroiditis. This is paralleled by weaker metabolic effects in men with thyroiditis than in men without thyroid pathology. Thus, men with thyroid autoimmunity may benefit to a lesser degree from metformin treatment and may require specific therapy to lower titers of thyroid antibodies. These relationships seem similar to those in women, which suggests that the impact of autoimmune thyroiditis on metformin action is sex independent. Study limitations and the lack of other studies justify conducting a larger trial to verify the obtained results. 

## 4. Materials and Methods

This single-center, prospective cohort study followed the principles of the Declaration of Helsinki, and its protocol was accepted by the Bioethical Committee of the Medical University of Silesia (KNW/0022/KB/207/17; 17 October 2107). All participants gave written informed consent after receiving oral and written information about the study from the investigators. Due to its character, the study did not require registration at a clinical trial registry.

### 4.1. Study Population

The participants of the study were recruited among middle-aged or elderly men (50–75 years old) with antipsychotic-induced hyperprolactinemia who, because of recently diagnosed type-2 diabetes or prediabetes, required metformin treatment. We included only patients with the total prolactin concentration in the range between 30 and 80 ng/mL on two different occasions and with a difference between both measurements not exceeding 10%. The widely accepted American Diabetes Association criteria were used to diagnose type-2 diabetes (fasting glucose greater than or equal to 126 mg/dL or 2 h post-challenge glucose equal to 200 mg/dL or higher) or prediabetes (fasting glucose between 100 and 125 mg/dL and/or 2 h post-challenge glucose between 140–199 mg/dL). The participants were chosen only among individuals complying with lifestyle recommendations for at least 12 weeks. The participants were allocated into one of two groups, each consisting of 24 men. The number of included patients was based on the sample-size analysis showing that 22 patients per group would be sufficient to provide significant data (25% difference in total prolactin) for 80% power with 95% confidence. Recruitment of two additional patients in each group aimed to compensate for possible dropouts. Individuals recruited to group A had to fulfill the following criteria of euthyroid Hashimoto thyroiditis: (a) titers of thyroid peroxidase antibodies (TPOAb) exceeding 100 U/mL; (b) a hypoechoic, diffusely heterogeneous echotexture with hypoechoic micronodules and/or surrounding echogenic septation on the ultrasound image; (c) plasma TSH concentration ranging from 0.4 to 4.5 mU/L; (d) free thyroxine concentration in the range between 10.2 and 21.4 pmol/L and (e) free triiodothyronine concentration in the range between 2.2 and 6.7 pmol/L. In turn, patients recruited to group B, serving as a control group, were characterized by (a) normal levels of TSH and free thyroid hormones, (b) non-elevated thyroid antibody titers, and (c) no changes in the sonographic picture of the thyroid gland. The control patients were selected from a group of 60 men who met the inclusion and exclusion criteria in order to obtain two populations similar in terms of age, body mass index (BMI), homeostatic model assessment of insulin-resistance ratio (HOMA-IR), and total prolactin concentrations. Similar proportions of men were enrolled in May or June (25 patients: group A: 13 men and group B: 12 men), and in November or December (23 patients: group A: 11 men and group B: 12 men).

The potential participants were excluded if they had a prolactin excess of another origin, macroprolactinemia, positive antibodies against thyrotropin receptor, other autoimmune or endocrine diseases, cardiovascular disorders (except for grade-1 hypertension), an estimated glomerular filtration rate less than 60 mL/min/1.73 m^2^, liver failure, malabsorption syndromes, oncological disorders, any other serious disorders, received other medicines (except for antipsychotic drugs), or were poorly compliant.

### 4.2. Study Design

**Figure 1:**
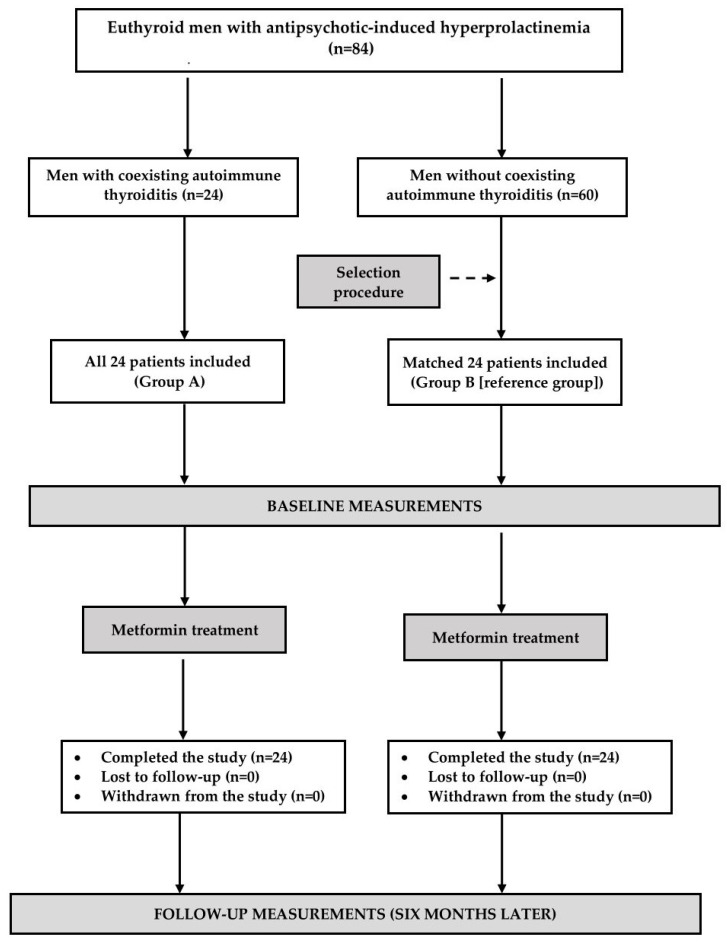
Flow the patients through the study.

The flow of patients through the study is depicted in Figure 1. The initial dosing for metformin was 500 mg by mouth twice daily. Daily dosing was then increased in 500 mg increments weekly to a target dose of 3 g (1 g three times a day). Throughout the study, antipsychotic dosing remained unchanged, and other treatments were not allowed. Non-pharmacological interventions (diet and physical activity) from before the study began were continued. Adherence to pharmacotherapy was assessed every two months by asking the patient and counting tablets.

### 4.3. Laboratory Assays

Blood samples for laboratory analysis were collected between 7.00 and 8.30 a.m. in a quiet and air-conditioned room (constant temperature of 23–24 °C). All assays were performed in duplicate on the first study day (before the first metformin dose) and 6 months later. Plasma levels of glucose, creatinine, and whole-blood content of glycated hemoglobin were measured using the multi-analyzer COBAS Integra 400 Plus (Roche Diagnostics, Basel, Switzerland). Titers of thyroglobulin antibodies (TgAb) and TPOAb and plasma concentrations of prolactin, insulin, thyrotropin, free thyroxine, free triiodothyronine FSH and LH were assessed using acridinium ester technology (ADVIA Centaur XP Immunoassay System, Siemens Healthcare Diagnostics, Munich, Germany). Circulating levels of adrenocorticotropic hormone (ACTH), insulin-like growth factor-1 (IGF-1), and high-sensitivity C-reactive protein (hsCRP) were measured by solid-phase enzyme-labeled chemiluminescent immunometric assays (Immulite, Siemens, Munich, Germany). Total prolactin was measured before, whereas monomeric prolactin was measured after polyethylene glycol precipitation [38]. Macroprolactin was calculated by subtracting monomeric prolactin from the total prolactin. Concentrations of adrenocorticotropic hormone (ACTH) and insulin-like growth factor-1 (IGF-1) were measured by solid-phase enzyme-labeled chemiluminescent immunometric assays (Immulite, Siemens, Munich, Germany). HOMA-IR was obtained by dividing the product of plasma glucose (in mg/dL) and insulin (in mIU/L) by 405. The estimated glomerular filtration rate was calculated as follows: 175 × [plasma creatinine (µmol/L) × 0.0113]^−1.154^ × age (years)^0.203^ (the Modification Diet in the Renal Disease Study equation).

### 4.4. Statistical Analysis

Prior to data analysis, all variables were subjected to log transformation to meet the criteria of normality and homogeneity. The significance of differences between means was determined by Student’s *t*-tests for independent samples (inter-group comparisons) or paired Student’s *t*-tests (intra-group comparisons). The χ^2^ test was used to compare dichotomous variables. Between-variable correlations were assessed using Pearson’s correlation coefficients (r). Values of *p* below 0.05 indicated a significant difference.

## Figures and Tables

**Table 1:** Baseline characteristics of both study groups.

Variable	Group A	Group B	*p*-Value
Number (n)	24	24	-
Age (years)	60 ± 8	62 ± 9	0.4200
Type-2 diabetes (%)/prediabetes (%)	50/50	54/46	0.8342
Smokers (%)/Number of cigarettes a day (n)/Duration of smoking (years)	42/11 ± 5/32 ± 10	46/10 ± 6/34 ± 11	0.7523
BMI (kg/m^2^)	24.9 ± 4.3	24.5 ± 4.8	0.7624
Systolic blood pressure (mmHg)	130 ± 15	128 ± 14	0.6352
Diastolic blood pressure (mmHg)	85 ± 5	84 ± 5	0.4918

Group A: males with iatrogenic prolactin excess and euthyroid Hashimoto disease. Group B: males with iatrogenic prolactin excess but without thyroid disease. Except for the percentages of smokers, patients with type-2 diabetes, and individuals with prediabetes, the data have been shown as the mean ± standard deviation. Abbreviation: BMI—body mass index.

**Table 2:** The impact of metformin treatment on the assessed variables.

Variable	Group A	Group B	*p*-Value *
Glucose (mg/dL) [70–99]			
Baseline	121 ± 11	123 ± 12	0.5502
Follow-up	112 ± 10	105 ± 10	0.0193
*p*-value **	0.0048	<0.0001	-
HOMA1-IR [<2.0]			
Baseline	4.2 ± 1.3	4.0 ± 1.2	0.5824
Follow-up	3.2 ± 1.0	2.1 ± 0.8	0.0001
*p*-value **	0.0045	<0.0001	-
Glycated hemoglobin [4.0–5.6]			
Baseline	6.7 ± 0.5	6.8 ± 0.6	0.5346
Follow-up	6.3 ± 0.5	5.9 ± 0.4	0.0037
*p*-value **	0.0080	<0.0001	-
Total prolactin (ng/mL) [5–17]			
Baseline	55.2 ± 12.3	56.8 ± 13.8	0.6735
Follow-up	52.7 ± 13.4	44.8 ± 11.8	0.0354
*p*-value **	0.5702	0.0022	-
Monomeric prolactin (ng/mL) [3–15]			
Baseline	50.4 ± 11.2	52.5 ± 12.9	0.5500
Follow-up	48.6 ± 10.6	40.9 ± 11.6	0.0216
*p*-value **	0.5918	0.0020	-
Macroprolactin (ng/mL) [2–12]			
Baseline	4.8 ± 3.1	4.3 ± 2.9	0.5667
Follow-up	4.1 ± 3.5	3.9 ± 2.5	0.8208
*p*-value **	0.4670	0.6112	-
TPOAb (IU/mL) [<35]			
Baseline	840 ± 305	13 ± 12	<0.0001
Follow-up	668 ± 285	11 ± 14	<0.0001
*p*-value **	0.0978	0.5978	-
TgAb (IU/mL) [<35]			
Baseline	828 ± 320	17 ± 12	<0.0001
Follow-up	685 ± 276	16 ± 18	<0.0001
*p*-value **	0.1042	0.8219	-
TSH (mIU/L) [0.4–4.5]			
Baseline	3.2 ± 1.3	3.0 ± 1.4	0.6105
Follow-up	2.8 ± 1.2	2.3 ± 1.2	0.1557
*p*-value **	0.2738	0.0693	-
Free thyroxine (pmol/L) [10.2–21.4]			
Baseline	14.8 ± 2.4	15.2 ± 2.6	0.5824
Follow-up	15.1 ± 2.7	15.6 ± 2.9	0.5395
*p*-value **	0.6860	0.6174	-
Free triiodothyronine (pmol/L) [2.2–6.7]			
Baseline	3.6 ± 0.8	3.5 ± 0.9	0.6860
Follow-up	3.7 ± 0.8	3.7 ± 1.0	1.0000
*p*-value **	0.6670	0.4701	-
FSH (U/L) [1.5–9.5]			
Baseline	3.4 ± 1.0	3.2 ± 1.2	0.5346
Follow-up	3.7 ± 1.3	3.6 ± 1.4	0.7988
*p*-value **	0.3749	0.2934	-
LH (U/L) [1.5–8.5]			
Baseline	2.9 ± 0.8	3.0 ± 1.4	0.7626
Follow-up	3.2 ± 1.0	3.6 ± 1.1	0.1940
*p*-value **	0.2571	0.1056	-
Testosterone (ng/mL) [3.5–17.0]			
Baseline	4.4 ± 1.0	4.2 ± 1.3	0.5532
Follow-up	4.7 ± 1.2	4.9 ± 1.5	0.6125
*p*-value **	0.3517	0.908	-
ACTH (pg/mL) [15–70]			
Baseline	29 ± 14	34 ± 18	0.2883
Follow-up	35 ± 15	38 ± 16	0.5061
*p*-value **	0.1587	0.4200	-
IGF-1 (ng/mL) [50–180]			
Baseline	102 ± 48	97 ± 40	0.6968
Follow-up	114 ± 50	118 ± 60	0.8030
*p*-value **	0.4007	0.1604	-
hsCRP (mg/L) [<1.5]			
Baseline	2.8 ± 1.0	2.2 ± 0.8	0.0263
Follow-up	2.6 ± 0.8	1.5 ± 0.6	<0.0001
*p*-value **	0.4481	0.0013	-
Estimated glomerular filtration rate (mL/min/1.73 m^2^) [<60]			
Baseline	89 ± 13	91 ± 15	0.6239
After 6 months	92 ± 14	92 ± 16	1.0000
*p*-value **	0.4457	0.8242	-

* Group A vs. Group B, ** Follow-up vs. baseline. Group A: males with iatrogenic prolactin excess and euthyroid Hashimoto disease. Group B: males with iatrogenic prolactin excess but without thyroid disease. Except for the percentage of smokers, the data have been shown as the mean ± standard deviation. Reference values are shown in square brackets. Abbreviations: ACTH—adrenocorticotropic hormone; FSH—follicle-stimulating hormone; HOMA-IR—the homeostatic model assessment of insulin-resistance ratio; hsCRP—high-sensitivity C-reactive protein; IGF-1—insulin growth factor-1; LH—luteinizing hormone; TgAb—thyroglobulin antibodies; TPOAb—thyroid peroxidase antibodies; TSH—thyroid-stimulating hormone.

**Table 3:** Percentage changes from baseline in the investigated variables during metformin treatment.

Variable	Group A	Group B	*p*-Value
Δ Glucose	−7 ± 3	−15 ± 6	<0.0001
Δ HOMA1-IR	−24 ± 20	−48 ± 23	0.0004
Δ Glycated hemoglobin	−6 ± 5	−13 ± 5	<0.0001
Δ Total prolactin	−5 ± 8	−21 ± 8	<0.0001
Δ Monomeric prolactin	−3 ± 7	−22 ± 10	<0.0001
Δ Macroprolactin	−15 ± 18	−9 ± 20	0.2803
Δ TPOAb	−20 ± 18	−15 ± 24	0.4184
Δ TgAb	−17 ± 23	−6 ± 28	0.1438
Δ TSH	−13 ± 14	−23 ± 22	0.0667
Δ Free thyroxine	2 ± 6	3 ± 7	0.5988
Δ Free triiodothyronine	3 ± 10	6 ± 14	0.3974
Δ FSH	9 ± 10	13 ± 12	0.2160
Δ LH	10 ± 12	20 ± 23	0.0653
Δ Testosterone	7 ± 13	17 ± 20	0.0457
Δ ACTH	21 ± 20	12 ± 18	0.1081
Δ IGF-1	12 ± 25	22 ± 20	0.1328
Δ hsCRP	−7 ± 14	−32 ± 20	<0.0001
Δ Estimated glomerular filtration rate	3 ± 7	1 ± 6	0.2934

Group A: males with iatrogenic prolactin excess and euthyroid Hashimoto disease. Group B: males with iatrogenic prolactin excess but without thyroid disease. Except for the percentage of smokers, the data have been shown as the mean ± standard deviation. Abbreviations: ACTH—adrenocorticotropic hormone; FSH—follicle-stimulating hormone; HOMA-IR—the homeostatic model assessment of insulin-resistance ratio; hsCRP—high-sensitivity C-reactive protein; IGF-1—insulin growth factor-1; LH—luteinizing hormone; TgAb—thyroglobulin antibodies; TPOAb—thyroid peroxidase antibodies; TSH—thyroid-stimulating hormone.

## Data Availability

The data that support the findings of this study are available from the corresponding author upon reasonable request.

## Abbreviations

ACTH—adrenocorticotropic hormone; AMPK—adenosine 5′-monophosphate-activated protein kinase; FSH—follicle-stimulating hormone; HOMA-IR—homeostatic model assessment of insulin-resistance ratio; hsCRP—high-sensitivity C-reactive protein; IGF-1—insulin-like growth factor-1; LH—luteinizing hormone; TgAb—thyroglobulin antibodies; TPOAb—thyroid peroxidase antibodies; TSH—thyroid-stimulating hormone.
